# Comparison of Reproductive Function Between Normal and Hyperandrogenemia Conditions in Female Mice With Deletion of Hepatic Androgen Receptor

**DOI:** 10.3389/fendo.2022.868572

**Published:** 2022-06-09

**Authors:** Mingxiao Feng, Sara Divall, Dustin Jones, Vaibhave Ubba, Xiaomin Fu, Ling Yang, Hong Wang, Xiaofeng Yang, Sheng Wu

**Affiliations:** ^1^ Department of Pediatrics, Johns Hopkins University School of Medicine, Baltimore, MD, United States; ^2^ Department of Pediatrics, Seattle’s Children’s Hospital, University of Washington, Seattle, WA, United States; ^3^ Department of Cellular and Molecular Physiology, Johns Hopkins University School of Medicine, Baltimore, MD, United States; ^4^ Department of Cardiovascular Sciences/Center for Metabolic Disease Research, Temple University School of Medicine, Philadelphia, PA, United States; ^5^ Department of Endocrinology, The First Medical Center, Chinese PLA General Hospital, Beijing, China; ^6^ Medical Genetics and Molecular Biochemistry, Temple University School of Medicine, Philadelphia, PA, United States

**Keywords:** PCOS: polycystic ovary syndrome, DHT, dihydrotestosterone, androgen receptor (AR), liver, puberty

## Abstract

Obesity, altered glucose homeostasis, hyperinsulinism, and reproductive dysfunction develops in female humans and mammals with hyperandrogenism. We previously reported that low dose dihydrotestosterone (DHT) administration results in metabolic and reproductive dysfunction in the absence of obesity in female mice, and conditional knock-out of the androgen receptor (Ar) in the liver (LivARKO) protects female mice from DHT-induced glucose intolerance and hyperinsulinemia. Since altered metabolic function will regulate reproduction, and liver plays a pivotal role in the reversible regulation of reproductive function, we sought to determine the reproductive phenotype of LivARKO mice under normal and hyperandrogenemic conditions. Using Cre/Lox technology, we deleted the *Ar* in the liver, and we observed LivARKO female mice have normal puberty timing, cyclicity and reproductive function. After DHT treatment, like control mice, LivARKO experience altered estrous cycling, reduced numbers of corpus lutea, and infertility. Liver Ar is not involved in hyperandrogenemia-induced reproductive dysfunction. The reproductive dysfunction in the DHT-treated LivARKO lean females with normal glucose homeostasis indicates that androgen-induced reproductive dysfunction is independent from metabolic dysfunction.

## Introduction

Hyperandrogenism in females is associated with metabolic and reproductive dysfunction in humans (1) and various animal models (2). The reproductive dysfunction of hyperandrogenic females includes irregular ovarian cyclicity and follicle development, and infertility (2). The metabolic dysfunction includes systemic insulin resistance, increased fat mass associated with adipocyte hypertrophy (3) and altered liver function. Liver plays important role in the reversible regulation of reproductive function (4). The liver controls energy storage and balance through directing glucose metabolism; this control of energy balance can thus regulate the reproductive axis which is sensitive to changes in systemic energy balance.

Androgen receptor (AR) signaling is present in liver involved in systemic glucose regulation (5–8). Female mice with global deletion of the Ar do not experience the metabolic and reproductive dysfunction upon androgen treatment that is experienced by wild type mice (9). This indicates the role of the Ar in both the metabolic and reproductive phenotype of hyperandrogenic females. Hepatocyte Ar is not required for glucose homeostasis in female mice with normal androgen levels (10), but plays a critical role in hepatocyte dysfunction associated with hyperandrogenism in females (11). LivARKO mice treated with low dose dihydrotestosterone (DHT) did not experience the hepatic insulin resistance/upregulated gluconeogenesis like DHT treated control mice (11). Since altered metabolic function may affect reproduction, and the liver and the reproductive system interacts in a complex bidirectional fashion, we sought to determine the reproductive phenotype of LivARKO mice under normal and hyperandrogenemia conditions. We explore the reproductive phenotype of female LivARKO at baseline and upon treatment with low dose DHT – an environment wherein the LivARKO mice maintain normal glucose homeostasis.

## Materials and Methods

### Generation of Liver Specific AR Knockout and Hyperandrogenemic Females

Hepatocyte Ar knockout mice were maintained in our laboratory as previously described (11). Briefly, we crossed an exon 2-floxed *Ar* (12, 13) female (AR^fl/fl^; Cre^-/-^) mouse, with a male albumin-*Cre* ^+/-^ mouse to produce developmental hepatic Ar knockout mice (LivARKO, AR^fl/fl^; albumin (Alb)-Cre^+/-^), The Alb-Cre mouse produces liver-specific expression of Cre driven by the albumin promoter in a C57/BL6 background. Litter mates (AR^fl/fl^; Alb-Cre^-/-^) were referred to as Control (Con) mice. Genotyping primers were designed to detect the presence of *Cre* or the floxed allele, WT allele, or knockout allele of *Ar*. Genomic DNA obtained from tail or ear was used for genotyping. Genomic DNA obtained from the liver will amplify a 952-bp amplicon for floxed *Ar* allele, if the sequence between the LoxP sites is excised, it will amplify a 404-bp amplicon for *Ar* KO allele. Primer sets were listed in **Supplementary Table 1**. Once female mice reached 2-months old, 4 mm-DHT (DHT) or vehicle (Veh) pellets were inserted to the mice under the skin (14–19). Two months after insertion of the pellets, body weight was recorded. All mice were housed in the Johns Hopkins University mouse facility, and all experiments were conducted under a protocol approved by the Johns Hopkins Animal Care and Use Committee.

### qRT-PCR

RNA isolation was performed on collected tissues from Con-veh and LivARKO-veh mice using Trizol (BioRad) as previous described (20). *Ar* mRNA levels in liver, ovary, uterus, hypothalamus, pituitary, gonadal fat and muscle were measured by quantitative real-time PCR (qRT-PCR) using iQSYBR green reagent according to the manufacturer’s protocol (Bio-Rad). Briefly, 1µg of RNA was reverse transcribed to cDNA using an iScript cDNA kit (Bio-Rad Laboratories). Real-time qPCR was performed to determine the presence and relative expression levels of *Ar* mRNA in the various tissues. Real-time qPCR was performed in duplicate using SYBR Green Master Mix (Bio-Rad Laboratories) and the CFX Connect qPCR machine (Bio-Rad Laboratories). For *Ar* primer set (listed in **Supplementary Table 1**), PCR efficiency was determined by measuring a 10-fold serial dilutions of cDNA and reactions having 95% and 105% PCR efficiency were included in subsequent analyses. Relative differences in cDNA concentration between WT and LivARKO mice were then calculated using the comparative threshold cycle (Ct) method. To compare the difference of *Ar* expression in the same tissue between WT and LivARKO, a △Ct was calculated to normalize for internal control using the equation: Ct (A*r*) – Ct (18S). △△Ct was calculated: △Ct (LivARKO) -△Ct (WT). Relative *Ar* mRNA levels were then calculated using the equation fold difference = 2△△Ct.

### Assessment of Puberty, Estrous Cyclicity and Reproductive Phenotypes in LivARKO Females

Puberty was assessed beginning at 21 days of age by visual inspection of vaginal opening and assessing the age of first estrus (21). Estrous cyclicity was determined, beginning at 8 weeks of age and continuously for 24 days, by assessing vaginal cytology (21). Fertility was assessed by mating 2-3 month old female mice with proven fertile wild type male mice for 90 days, and recording the number of pups and number of litters per female (21, 22). The examiners were blinded to genotypes during all data collection.

### Analyzing Estrous Cyclicity and Reproductive Phenotypes in Hyperandrogenemic Females

Females implanted with DHT (Con-DHT vs LivARKO-DHT) or an empty pellet (Con-veh vs LivARKO-veh) for 15 days were divided into two groups. Group 1 underwent examination of estrous cyclicity by vaginal cytology for 24 days starting at day 15 after DHT treatment. Females in group 2 were mated with fertile males (one female with one male per cage) for 90 days starting at day 15 of DHT treatment. Fertility was examined as described above.

### Histology Assays

The ovary was dissected from diestrus mice and fixed in 10% formalin phosphate buffer and sectioned to 5 microns thickness in its entirety by Johns Hopkins Medical Laboratories (Histology group). Every 10th section was collected, and ovarian sections were stained with hematoxylin and eosin and examined with a Zeiss microscope.

### Hormone Assays

Blood samples were collected from submandibular vein (17, 21) between 9:00 and 10:00 AM and basal levels of serum LH and FSH were measured. LH and FSH from serum of mice at diestrus were measured by Luminex assay ((MPTMAG-49K, Millipore, Billerica, MA) on a Luminex 200IS platform (Luminex Corporation). The assay detection limit for LH was 0.012 ng/mL and for FSH was 0.061ng/mL. The intra-assay and interassay coefficients of variation (CV) for LH and FSH were between 5% and 9%.

### Glucose Tolerance Test

Mice were fasted overnight (16 h) and received intraperitoneal (i.p.) injections of 2 g/kg body weight (BW) glucose. Glucose level was measured at 0, 15, 30, 60, 90 and 120 minutes after glucose injection.

### Statistical Analysis

Statistical analyses used were described in each individual figure legend. Some data were analyzed by student t-tests. Some data were assessed by 2-way ANOVA with main effects of DHT treatment and genotype assessed. All analyses were performed using Prism software (GraphPad, Inc.). All results were expressed as means ± SEM. A value of p<0.05 was defined as statistically significant.

## Results

### Deletion of Androgen Receptor Specifically in Liver

**Figure 1:**
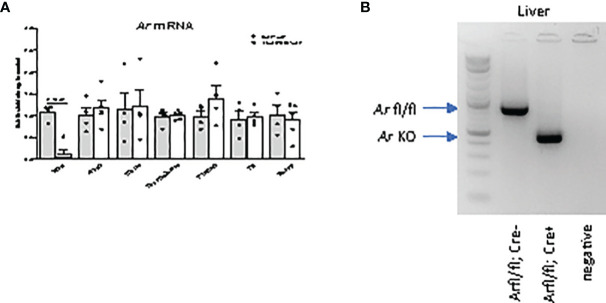
Androgen receptor (*Ar*) is specifically deleted in liver. **(A)** *Ar* mRNA levels of LivARKO compared to control littermates, measured by qRT-PCR, in liver, ovary, uterus, hypothalamus, pituitary, gonadal fat, and skeletal muscles. **(B)** PCR analysis of *Ar* liver genomic DNA from control and LivARKO mouse. Arrows pointed to a 952 bp *Ar* floxed band in liver of control mouse and a knockout band 404 bp in liver of LivARKO mouse. Two-tailed student’s t-tests were applied. P-values were stated in the graphs. N=4-5. Values were mean ± S.E.M.

A hepatocyte-specific AR knockout mouse was generated using the CRE/lox system (11). In LivARKO mice, *Ar* mRNA expression in the liver was significantly reduced (90%) compared to control mice littermates, while *Ar* mRNA expression was not altered in the ovaries, uterus, brain, pituitary, fat or muscle (**Figure 1A**). The PCR product from liver DNA indicated the homozygous floxed-*Ar* alleles in control mouse and KO alleles in LivARKO mouse (**Figure 1B**). In our observations (11, 15, 17) and others (10, 23), mice with AR^fl/fl^, Alb-Cre ^-/-^ do not exhibit a different reproductive or metabolic phenotype, thus AR^fl/fl^ mice are commonly used as control mice.

### Female LivARKO Mice Have Normal Puberty, Estrous Cycling and Fertility

**Figure 2:**
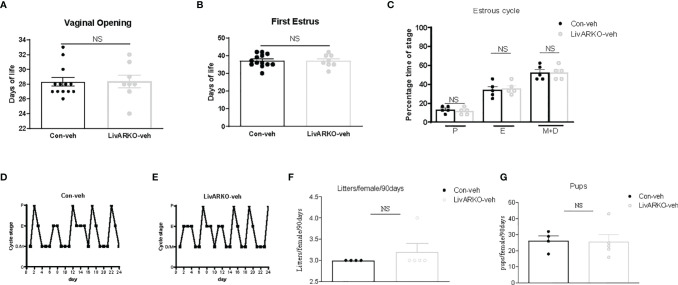
Female puberty, cyclicity and fertility. LivARKO mice exhibited similar age of puberty onset, assessed by examination of vaginal opening **(A)** and first estrus **(B)**, as control mice. **(C)**. Percentage of time spent in each of the stages (4-month old) was not significantly different between control and LivARKO mice. Representative data for vaginal cytology from individual Con **(D)**, and LivARKO mice **(E)**. There were no significant differences between LivARKO and controls in either total numbers of litters per female **(F)**, or numbers of pups per female **(G)**. Data were compared by two-tailed student’s t-tests. Values are mean ± SEM, n = 5-13/group. NS, non-significant; P, proestrus; E, estrus; M, metestrus; D, diestrus.

To assess age of pubertal onset in female mice, age of initial vaginal opening and first estrus was determined. Compared to control littermates, LivARKO females experienced vaginal opening (28.3 ± 0.6 vs 28.4 ± 0.9 day of life) and first estrus (37.1 ± 1.0 vs 37.0 ± 1.3 day of life) at similar ages (**Figures 2A, B**). There was also no difference in ovarian estrous cycle duration or pattern (**Figures 2C–E**); LivARKO female mice spent similar amounts of time in proestrus, estrus, and met/diestrus as control mice. During the mating period of 90 days, there was no significant difference between control and LivARKO mice in either the number of litters or number of pups per female (**Figures 2F, G**).

### Female LivARKO-DHT Mice Have Altered Cyclicity and Fertility

**Figure 3:**
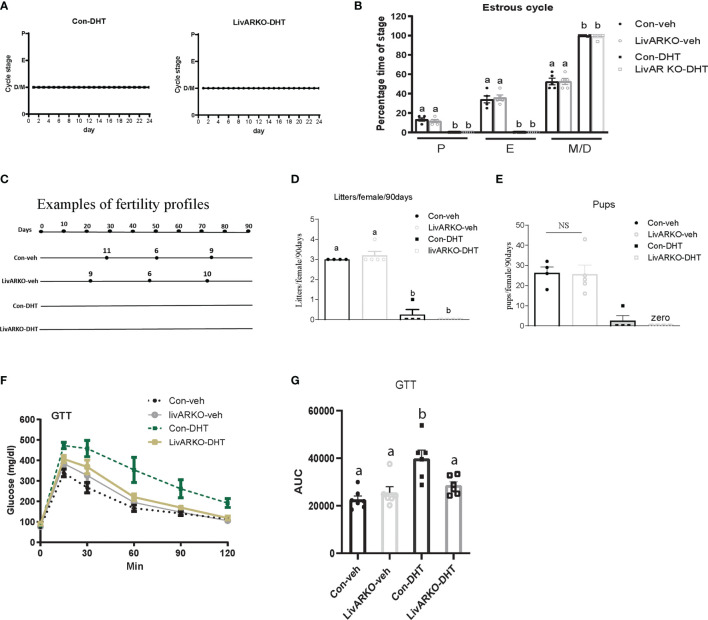
Cyclicity and fertility of mice with DHT treatment. Representative data for vaginal cytology from individual Con-DHT and LivARKO-DHT mice **(A)**. **(B)** Estrous cycles. Percentage time spent in each stage of the estrous cycle was significantly differently among the four groups. Metestrus (M)/diestrus **(D)** was shown consistently in DHT treated mice. **(C)** Example of fertility profile: female mice were mated with wild type male mice for 90 days. Each line represents an individual female mouse of each group. The black dot represents the day that each litter was born after introduction to male. Number on the top of line represents how many pups in each litter. Total numbers of litters **(D)** and pups **(E)** per female were significantly reduced in DHT treated (Con-DHT and LivARKO-DHT) mice compared to vehicle treated mice during the 90 days of mating. **(F)** Control and LivARKO mice, with and without DHT treatment were subjected to glucose tolerance test (GTT). **(G)** The area under the curve (AUC) was determined for the GTT. Statistical analysis was performed using two-way ANOVA followed by Tukey’s multiple tests. Bars with different letters represent significantly different values from each other with p<0.05. Values are mean ± SEM, n = 4-7/group.

We have previously reported that low dose DHT to control female mice results in reproductive and metabolic abnormalities (11, 15, 17) and that LivARKO mice do not experience the insulin resistance and glucose intolerance present in control mice upon DHT treatment (11, 19). We examined whether female LivARKO mice, while not exhibiting the metabolic derangements, would experience reproductive dysfunction. In both control and LivARKO vehicle treated mice (**Figure 2C–E**), estrous cycling was disrupted (consistent diestrus) upon treatment with DHT (**Figure 3A**). Estrous cycles in the 4 groups of mice were shown in **Figure 3B**.

Examples (one mouse as representation per group) of fertility profiles are plotted for Con-DHT and LivARKO-DHT mice in **Figure 3C**. Number of litters and average litter size was compared among experimental groups ((litters: 3.0 ± 0.0 (Con-veh); 3.2 ± 0.2 (LivARKO-veh); 0.3 ± 0.3 (Con-DHT); 0.0 ± 0.0 (LivARKO-DHT); number of pups: 26.3 ± 3.0; 25.6 ± 4.6; 2.5 ± 2.5; 0.0 ± 0.0)) and graphed in**Figure 3D, E**. Vehicle treated data are displayed as references. The number of litters (**Figure 3D**) and pups per female (**Figure 3E**) of LivARKO-DHT and Con-DHT mice during the mating period was significantly reduced compared to Con-veh and LivARKO-veh mice. In the presence of hyperandrogenemia, LivARKO-DHT mice failed to exhibit impaired glucose tolerance compared to Con-DHT mice (**Figures 3F, G**).

### Female LivARKO-DHT Mice Have Similar Body Weight, CL, and LH and FSH Levels Compared to Con-DHT Mice

**Figure 4:**
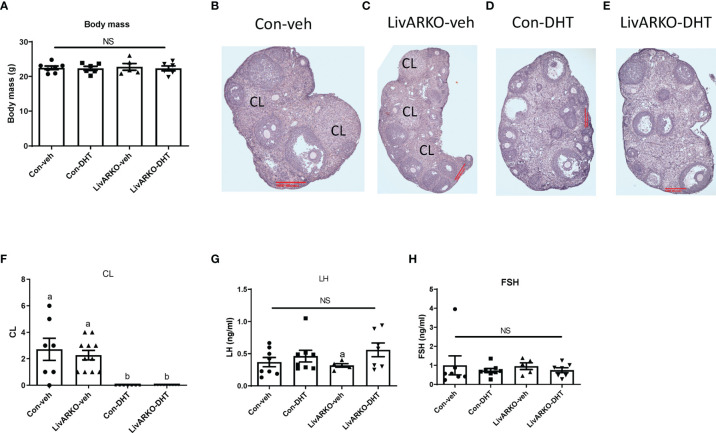
Body mass, ovarian structure, and LH and FHS levels of mice with DHT treatment. **(A)** Body mass was recorded at 4 month old. Representative ovary structure for Con-veh **(B)**, LivARKO-veh **(C)**, Con-DHT **(D)** and LivARKO-DHT **(E)**. **(F)** Corpora lutea (CL) was counted every 10th section per ovary (n=7-11 per group). LH **(G)** and FSH **(H)** levels were measured at basal (Met/Destrus) stage. There was no difference in body mas, CL, basal LH and FSH levels among groups. **(F)** Statistical analysis was performed using two-way ANOVA followed by Tukey’s multiple tests. Bars with different letters represent significantly different values from each other with p<0.05. Values are mean ± SEM, n = 5-11/group. NS: non-significant.

During the two months of DHT treatment, body mass (**Figure 4A**) was not altered among groups. Morphology of ovaries from Con-veh, LivARKO-veh, Con-DHT and LivARKO-DHT mice is shown in **Figures 4B–E**. Ovarian architecture was similarly altered upon DHT treatment in both control and LivARKO mice (**Figures 4D, E**). The most marked difference was abundance of the CL ((corpora lutea: 2.7 ± 0.8 (Con-veh); 2.3 ± 0.4 (LivARKO-veh); 0.0 ± 0.0 (Con-DHT); 0.0 ± 0.0 (LivARKO-DHT)) which were much less common in the ovaries of the DHT treated mice (**Figures 4D–F**) than in any of the vehicle treated groups (**Figures 4B, C, F**). There was no significant difference in serum levels of LH and FSH among Con-veh, LivARKO-veh, Con-DHT and LivARKO-DHT groups: LH: 0.36 ± 0.07 vs 0.46 ± 0.09 vs 0.32 ± 0.03 vs 0.56 ± 0.10ng/ml; FSH 0.99 ± 0.50 vs 0.72 ± 0.11 vs 0.95 ± 0.18 vs 0.74 ± 0.13ng/ml (**Figures 4G, H**).

## Discussion

Multiple organs and hormones are involved in the pathophysiology of androgen-induced metabolic and reproductive dysfunction. Liver dysfunction encompassing hepatocyte insulin resistance and up regulated gluconeogenesis occurs in hyperandrogenism in the lean and obese state. We were able to delete *Ar* in liver but not in other tissues at mRNA levels (**Figure 1**) in addition to protein levels (11). Hepatocyte Ar does not play an essential role in female reproductive function in the normal and hyperandrogenic state as puberty timing, estrous cycling, and fertility were similar in the LivARKO and control mice under normal and hyperandrogenic conditions.

The LivARKO female mice have similar weights as control mice, consistent with previous findings (10). LivARKO mice also have normal hepatic and peripheral glucose handling (10, 11), an environment that is associated with normal reproductive function so it is not surprising that they have reproductive function similar to control mice under physiological androgen conditions. Because of the role of Ar in organ dysfunction in the hyperandrogenic state, we next induced hyperandrogenemia in the LivARKO mice. Upon treatment with DHT, LivARKO mice exhibit reproductive dysfunction with altered estrous cycling, and infertility to a similar degree (**Figures 3**, **4**) as control mice. Importantly, LivARKO mice treated with DHT do not experience disrupted metabolic changes (**Figures 3F–G**) as previously reported (11). That reproductive dysfunction occurs in the DHT treated LivARKO mice with normal hepatic glucose homeostasis suggests that androgen-induced reproductive dysfunction can occur independently of androgen-induced metabolic dysfunction.

It is beyond the scope of this paper to explore the mechanism underlying the reproductive dysfunction in the DHT- treated control and LivARKO mice. Global AR knockout mice treated with DHT do not experience changes in body composition, ovarian or adipocyte dysfunction like wild type mice, indicating that the Ar plays a role in these tissues (9). The Ar of the ovarian theca cells (15), pituitary gonadotrope (17), and the neuron (24) all play a role in the reproductive dysfunction of hyperandrogenic females.

In the DHT treated control or LivARKO mice, the LH is not significantly different from untreated mice (**Figure 4**). This observation is like other rodent models of hyperandrogenemia (24, 25) and different than women with hyperandrogenism and polycystic ovary syndrome (PCOS), who can experience a higher LH or LH/FSH ratio (26). Indeed, the LH pulse frequency is not different between DHT treated and untreated mice (25). In this way postnatal DHT mouse model of hyperandrogenemia does not completely recapitulate human PCOS which is a limitation of this study. However, the fact that LH is not elevated allows us to tease out the effect of hyperandrogenism on gonad function separate from any effect due to LH hypersecretion.

Our data significantly indicate that the liver AR does not play a critical role in the reproductive dysfunction associated with hyperandrogenemia in lean conditions, and that hyperandrogenemia affects reproductive function even in the absence hepatic associated metabolic derangements. The LivARKO treated with DHT mouse model allows us to study the effects of hyperandrogenism on reproductive tissues *in vivo* in the absence of liver-induced metabolic dysfunction, a feature not present in other rodent models of female hyperandrogenism. Future study may focus on how hepatic AR affects fertility and metabolic function under obese hyperandrogenemic conditions.

## Data Availability Statement

The original contributions presented in the study are included in the article/**Supplementary Material**. Further inquiries can be directed to the corresponding author.

## Ethics Statement

The animal study was reviewed and approved by Johns Hopkins Animal Care and Use Committee.

## Author Contributions

MF, SD, and SW contributed to the conceptual design, performance of experiments, interpretation, and analysis of data, and writing and editing the manuscript. All other authors contributed to performing some of the experiments, analyzing the corresponding data, and reviewing and editing the manuscript.

## Funding

This work was supported by the National Institutes of Health (Grants R00-HD068130 04 and R01-HD09551201A1 to SW).

## Conflict of Interest

The authors declare that the research was conducted in the absence of any commercial or financial relationships that could be construed as a potential conflict of interest.

## Publisher’s Note

All claims expressed in this article are solely those of the authors and do not necessarily represent those of their affiliated organizations, or those of the publisher, the editors and the reviewers. Any product that may be evaluated in this article, or claim that may be made by its manufacturer, is not guaranteed or endorsed by the publisher.

## Abbreviations

*AR, androgen receptor*; PCOS: polycystic ovary syndrome; DHT, dihydrotestosterone; CAH, congenital adrenal hyperplasia; Con, control; Veh, vehicle; LH, luteinizing hormone
