# Supplementary material for: Comparison of Reproductive Function Between Normal and Hyperandrogenemia Conditions in Female Mice With Deletion of Hepatic Androgen Receptor
Source: Front Endocrinol (Lausanne). 2022 Jun 9;13:868572. doi: 10.3389/fendo.2022.868572 (PMC9218244; doi:10.3389/fendo.2022.868572)
Supplement: Supplementary file 1 [file Table_1.docx]

| **Suppl. Table 1**  Primer set |  |  |  |
| --- | --- | --- | --- |
| genotype Primer | *Cre* | F | CGACCAAGTGACAGCAATGCT |
|  |  | R | GGTGCTAACCAGCGTTTTCGT |
|  | *Ar* fl/fl | F-mAR28 | AGCCTGTATACTCAGTTGGGG |
|  |  | R-mAR29 | AATGCATCACATTAAGTTGATACC |
| Q-PCR primer | *Ar* | F | GGCGGTCCTTCACTAATGTCAACT |
|  |  | R | GAGACTTGTGCATGCGGTACTCAT |
